# Screening and identification of key serum biomarkers between PM2.5 and-induced asthma onset

**DOI:** 10.3389/fpubh.2025.1693575

**Published:** 2026-01-12

**Authors:** Yong Tang, Liangjun Li, Wenjie Hao, Hongyan Xu, Xiangyun Deng

**Affiliations:** 1 Department of Laboratory Medicine, Wuwei Traditional Chinese Medicine Hospital, Wuwei, Gansu, China; 2 Department of Respiratory Medicine, Jiayuguan First People’s Hospital, Jiayuguan, Gansu, China

**Keywords:** air pollution, asthma, mediation effect, PM2.5, genes

## Abstract

**Objective:**

The mechanism by which air pollution causes the onset of asthma is complex, and its key targets have not yet been fully identified. In this study, we identified the factors that mediate the relationship between air pollution and asthma.

**Methods:**

We screened overlapping genes related to asthma from the Gene Expression Omnibus (GEO) database by integrating differentially expressed genes (DEGs) and weighted gene co-expression network analysis (WGCNA). To further identify hub genes, we used three machine learning methods: least absolute shrinkage and selection operator (LASSO) regression, support vector machine-recursive feature elimination (SVM-RFE), and random forest (RF). We subsequently analyzed the mediating role of these hub genes in the association between PM2.5 and onset of asthma in a study population consisting of 160 participants with asthma and 160 participants without asthma. The analyses included Pearson correlation analysis, logistic regression analysis, and mediation analysis.

**Results:**

A total of 715 DEGs were identified, and the results of the WGCNA revealed a significant asthma-associated co-expression grey module containing 118 genes. Among these genes, five hub genes (CEBPE, HDC, IRAK3, PRR4, and SOD2) were selected using three different machine learning methods. These genes were confirmed as independent predictors of asthma through multivariate logistic regression analysis and were significantly correlated with PM2.5 levels. Mediation analysis demonstrated that these genes play a mediating role between PM2.5 and asthma onset.

**Conclusion:**

This study provided evidence that CEBPE, HDC, IRAK3, PRR4, and SOD2 mediate the connection between PM2.5 and the onset of asthma.

## 1 Introduction

Asthma is a common chronic inflammatory airway disease that is characterized primarily by recurrent narrowing and spasms of the respiratory tract, resulting in symptoms such as wheezing, shortness of breath, chest tightness, and coughing (1). The pathogenesis of asthma is quite complex and involves multiple factors. Among these factors, genetic factors are an important reason for its onset. Environmental factors also strongly influence the occurrence of the disease (2, 3). Epidemiological studies have reported that due to rapid industrialization and urbanization, the problem of air pollution has become increasingly severe. Long-term exposure to polluted air can increase the risk of respiratory diseases, especially asthma (4–6). The airway sensitivity of asthma patients who are exposed to polluted air for a long period may further increase. Even low-concentration irritants may trigger asthma attacks, increasing the frequency and severity of asthma attacks. Environmental pollution also encompasses exposure to allergens, respiratory tract infections, and climate change, among other factors (7, 8). These factors can interact with air pollution, further influencing the onset of asthma. However, the specific molecular mechanisms by which air pollution leads to the onset of asthma need to be elucidated.

To understand the intrinsic connection between environmental air pollution and the onset of asthma, many studies have been conducted (9, 10). Among these studies, the use of serum markers as intermediary variables has received considerable attention. Recently, Nahid Mostafavi et al. provides supportive evidence for a mediating effect of the immune system in the association between air pollution and adult-onset asthma (11). Furthermore, several studies also have investigated the significant role of genes in this process to understand how changes of gene expression interacts with environmental factors to jointly affect the occurrence and development of asthma (12, 13). From a prospective observational cohort study and a randomized, double-blind, placebo-controlled trial in an independent cohort, Matthew C Altman’s study demonstrated that individual pollutants were significantly associated with altered gene expression in coordinated inflammatory pathways, including PM2·5 with increased epithelial induction of tissue kallikreins, mucus hypersecretion, and barrier functions and O3 with increased type-2 inflammation (14). Therefore, the identification of mediators is helpful to the prevention of asthma onset and early warning of asthma attack. In addition, it also has guiding significance for formulating relevant public health policies, so as to better protect vulnerable populations.

In this study, we assessed the roles of genes in the association between PM2.5 and onset of asthma. For this, we used the Gene Expression Omnibus (GEO) database to identify hub genes dysregulated in asthma through differential expression gene (DEG) analysis, weighted gene co-expression network analysis (WGCNA), and machine learning methods. Data were collected from 160 participants with asthma and 160 participants without asthma. Univariate and multivariate logistic analyses were conducted to screen for factors influencing the onset of asthma. The mediating role of the hub genes in the association between PM2.5 and onset of asthma was subsequently evaluated using the bootstrap method. This study provided new theoretical insights into the pathogenesis of asthma and identified therapeutic targets for future intervention strategies.

## 2 Methods

### 2.1 Data collection and preprocessing

Gene expression datasets were retrieved from the GEO database.^1^ Three independent datasets (GSE65204, GSE134544, and GSE137268) were included in this study. The datasets were selected based on the following criteria: (1) publicly available in the GEO database; (2) derived from human subjects; (3) contained information on both asthma and healthy control groups; (4) included airway-relevant or immunologically informative sample types; (5) had well-defined sample sizes and annotations. The raw expression data were normalized and preprocessed using the limma package in R (version 4.2.1). Probes were annotated according to platform-specific annotation files, and duplicates were collapsed according to the median expression value. The dataset information was shown in Table 1.

**Table 1:** Information on the dataset.

Datasets	Time	Asthma group	Control group	Platforms	Organism	Experiment type
GSE65204	Jan 22, 2015	36	33	GPL14550	Homo sapiens	Array
GSE134544	Jul 19, 2019	46	21	GPL10558	Homo sapiens	Array
GSE137268	Sep 11, 2019	54	15	GPL6104	Homo sapiens	Array

### 2.2 Differential expression analysis

To identify differentially expressed genes (DEGs) between the asthma and control groups in the dataset GSE65204, we used the empirical Bayes method implemented in the limma package. Genes with an absolute log_2_-fold change (|log₂FC|) > 0.25 and *p* < 0.05 were considered to be significant. In order to screen more DEGs for subsequent analysis, we used *p* value instead of adjusted *p* values. DEGs were visualized using volcano plots and heatmaps generated using the ggplot2 and pheatmap packages, respectively.

### 2.3 Weighted gene co-expression network analysis

To identify gene modules associated with asthma, WGCNA was performed on the GSE137268 dataset using the WGCNA package. After filtering for the top 5,000 most variable genes based on median absolute deviation (MAD), a scale-free topology model was constructed. The soft-thresholding power was determined using the pickSoftThreshold function. Modules were identified by average linkage hierarchical clustering and dynamic tree cutting with a minimum module size of 30. Module-trait relationships were calculated by correlating module eigengenes with the status of asthma. Modules showing the strongest correlation were retained for downstream analysis.

### 2.4 Machine learning for biomarker selection

Three machine learning algorithms were applied to the 18 intersecting candidate genes using the GSE134544 dataset, including least absolute shrinkage and selection operator (LASSO) regression, support vector machine-recursive feature elimination (SVM-RFE), and random forest (RF). LASSO regression was performed using the glmnet package, with 10-fold cross-validation to select the optimal lambda. SVM-RFE was conducted using the caret package, and the top-ranked features based on cross-validation accuracy were retained. RF analysis was performed using the randomForest package, and genes with an importance score >1.5 were selected. Candidate genes identified using all three algorithms were considered to be robust diagnostic biomarkers.

### 2.5 Gene set enrichment analysis

To investigate the biological functions of the identified biomarkers, GSEA was performed on each gene using the GSE65204 dataset. For each gene, samples were divided into high-expression and low-expression groups based on median expression. KEGG pathway enrichment was conducted using the GSEA software (version 4.1.0), with 1,000 permutations. Significantly enriched pathways were defined by a nominal *p* < 0.05 and FDR < 0.25. Enrichment plots were generated to visualize the top-ranked pathways for each biomarker.

### 2.6 Participants

This study was conducted at Jiayuguan First People’s Hospital and approved by the Ethics Committee of Jiayuguan First People’s Hospital. All participants provided signed informed consent. From January 2021 to December 2024, 320 participants were signed up, including 160 participants with asthma and 160 participants without asthma. The study was conducted according to the Declaration of Helsinki, and its content and procedures complied with institutional ethical committee standards. This study was conducted after it was approved. Participants who met the criteria were recruited and evaluated. The inclusion criteria included having resided in the city for five consecutive years and having no history of other significant cardiopulmonary diseases. All participants voluntarily provided signed informed consent. The exclusion criteria were patients with pulmonary nodules, bronchiectasis, chronic obstructive pulmonary disease, chronic bronchitis, cystic fibrosis, or lung cancer; individuals with altered mental status, consciousness disorders, or other psychiatric conditions; those with hematologic malignancies; and individuals unwilling to participate in the study or those with incomplete clinical data. The patient meets the diagnostic criteria of the Guidelines for bronchial asthma prevent and management (2020 edition) (15). The diagnostic criteria included: ① Repeated wheezing, coughing, shortness of breath and chest tightness are often associated with exposure to allergens, cold air, physical and chemical substances. It is related to irritation, respiratory tract infection, exercise and hyperventilation, etc., and often occurs or worsens at night and/or in the early morning. ② During an attack, scattered or diffuse wheezing sounds mainly in the expiratory phase can be heard in both lungs, with the expiratory phase prolonged. ③ The above symptoms and signs are effective with anti-asthma treatment or resolve spontaneously. ④ Excluding wheezing, coughing, shortness of breath and chest tightness caused by other diseases. For those with atypical clinical manifestations (such as no obvious wheezing or wheezing sounds), at least one of the following should be present: (1) Confirmed reversible airflow limitation: (a) Positive bronchial dilation test: Fifteen minutes after inhaling a fast-acting ß2 receptor agonist (such as salbutamol pressure quantified aerosol 200–400 μg), the forced expiratory volume in the first second (FEV1) increased by more than 12% and its absolute value increased by more than 200 mL. (b) Improvement of pulmonary ventilation function after anti-inflammatory treatment: After 4 to 8 weeks of treatment with inhaled glucocorticoids and/or anti-leukotriene drugs, FEV1 increases by more than 12%; (2) Positive bronchial provocation test; (3) The daytime variation rate or diurnal fluctuation rate of peak expiratory flow (PEF) is greater than 20%. Those who meet the conditions of items 1–4 or items 4 and 5 can be diagnosed with asthma.

### 2.7 Air pollution status

This study referred to the basic standards specified in the “Environmental Air Quality Standard GB3095-2012” and obtained monitoring data of particulate matter 2.5 (PM2.5) from the local environmental monitoring department for the residential areas of the participants within the corresponding period. The risk of asthma in this region was assessed based on the obtained air pollutant concentrations (16).

### 2.8 Blood sample collection

Blood samples (5 mL) from patients with asthma were collected in vacuum coagulant tubes within 1 day of asthma exacerbation. We collected 5 mL of fasting elbow vein blood from participants without asthma in the morning. After centrifugation at 4 °C for 15 min at 3,000 rpm, serum samples were collected in RNase/DNase-free tubes and immediately frozen at −80 °C.

### 2.9 RNA extraction and quantitative real-time polymerase

Total RNA was extracted from the serum samples using TRIzol reagent (Takara, Dalian, China) following the manufacturer’s protocol. Then, the RNA was reverse-transcribed to cDNA using a PrimerScript RT-PCR kit (Takara). Real-time qPCR was performed using a standard SYBR Green PCR kit (Takara) following the manufacturer’s protocol. The relative expression of genes was calculated using the 2^–ΔΔCt^ method (17). GAPDH was used as an internal control. The sequences of primers used were as follows: for CCAAT/enhancer binding protein *ε* (CEBPE), forward: 5′- ATC TCT TTG CCG TGA AGC CA − 3′, reverse: 5′- TCT GCT GCG TCT CCA GAA TG − 3′; for histidine decarboxylase (HDC), forward: 5′- CCA TCT GTG CCC GTG AGG-3′, reverse: 5′- CGA AAA ACC ACC AGG CCA AG-3′; for interleukin-1 receptor-associated kinase 3 (IRAK3), forward: 5′- AGG ATT TCC GCG GTT GTG TA-3′, reverse: 5′- TCG ATG TCC CAT CTC CT-3′; for proline-rich protein 4 (PRR4), forward: 5′-TGT GTC CTC ACC CAC TGT-3′, reverse: 5′- GAG AGT TGA CGG TGT CCT CG-3′. For manganese superoxide dismutase (SOD2), forward: 5′- TCT GGC CCA CTC ACA GG AG − 3′, reverse: 5′- CTC GGT GAC GTT CAG GTT GT − 3′; for glyceraldehyde-3-phosphate dehydrogenase (GAPDH), forward: 5′- GGA TTT GGT CGT ATT GGG CG − 3′, reverse: 5′- TCC CGT TCT CAG CCA TGT AG − 3′.

### 2.10 Mediation analysis

**Figure 1:**
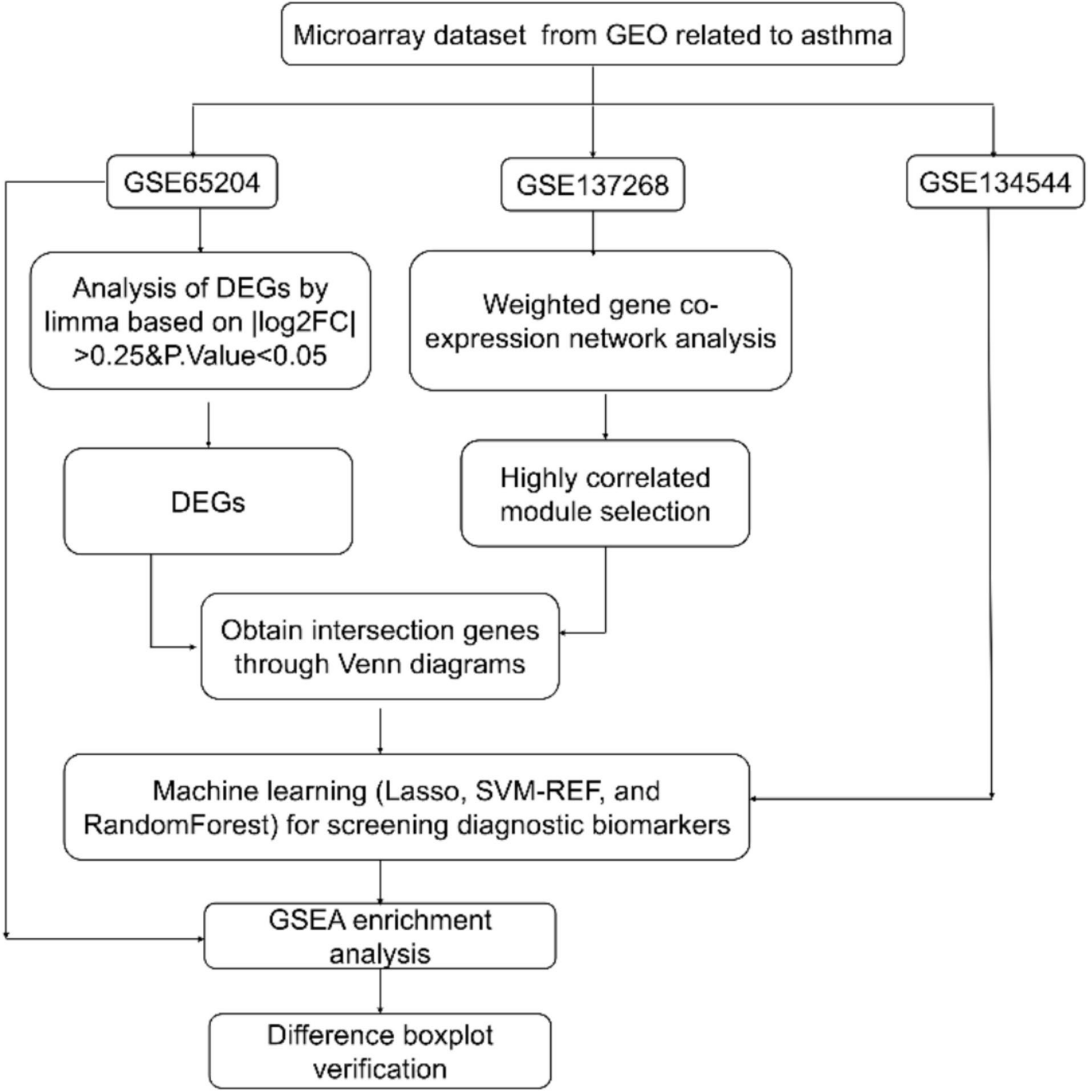
Overall flowchart of the study.

Mediation analysis was conducted usingR software with mediation package. First, a basic regression model was constructed with air pollution level as the independent variable and asthma incidence as the dependent variable, and the direct association between air pollution and asthma was analyzed. Then, genes were introduced into the model one by one, and their mediating effects were tested as mediator variables using bootstrap method with 1,000 bootstrap samples. When the Average Causal Mediation Effect (ACME) *p* < 0.05 and the Average Direct Effect (ADE) *p* > 0.05, it indicates a complete mediating effect. When the ACME p value < 0.05 and the ADE *p* < 0.05, it indicates a partial mediating effect. When the ACME *p* > 0.05 and the ADE *p* > 0.05, it indicates no mediating effect (Figure 1).

### 2.11 Statistical analysis

The data of the participants were compiled and analyzed using Statistical Product and Service Solutions (SPSS, IBM Corp., Armonk, NY, United States), version 23.0. The general data of the two groups were compared by conducting the Chi-square test or the Wilcoxon signed-rank test. The Pearson correlation coefficient was used to evaluate the relationship between the PM2.5 concentration and gene expression level. Univariate and multivariate logistic regression analyses were conducted to screen for factors related to asthma. All results were considered to be statistically significant at *p* < 0.05.

## 3 Results

### 3.1 Identification of differentially expressed genes

**Figure 2:**
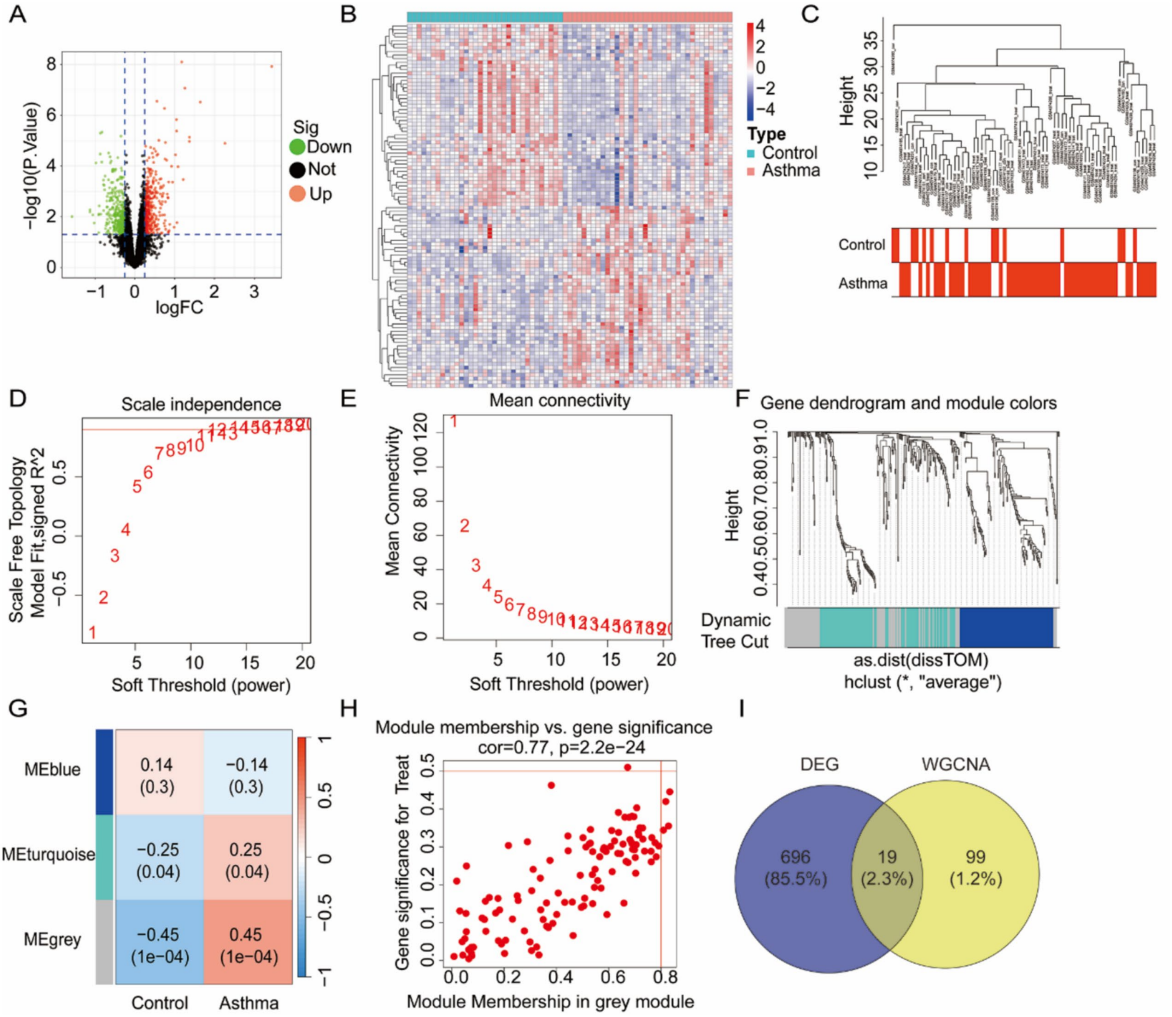
Differentially expressed genes (DEGs) and WGCNA of the GSE65204 dataset. **(A)** The volcano plot of GSE65204 shows DEGs with thresholds of |log_2_FC| > 0.25 and *p* < 0.05. Red, upregulated genes; green, downregulated genes; black, non-significant genes. **(B)** The heatmap illustrates the top 100 DEGs in GSE65204. **(C)** Sample dendrogram and trait heatmap. **(D,E)** Determination of soft-thresholding power based on scale-free topology (left) and mean connectivity (right). **(F)** Gene dendrogram with module colors after dynamic tree cut. **(G)** Module-trait relationships showed the correlation between module eigengenes and the status of asthma. **(H)** Identification of co-expression modules associated with asthma. **(I)** The Venn diagram shows 18 overlapping genes from the DEGs and WGCNA modules.

To identify DEGs between asthma patients and healthy controls, we analyzed the gene expression profiles from the GSE65204 dataset obtained from the GEO database. The DEGs were analyzed using the limma package, with the thresholds set at *p* < 0.05 and |log₂FC| > 0.25. In the GSE65204 dataset, 715 DEGs were identified, including 363 upregulated and 352 downregulated genes. A volcano plot was used to highlight these DEGs (Figure 2A), and the expression profiles of the top 100 DEGs were visualized using a heatmap (Figure 2B), which revealed distinct clustering between the asthma and control samples.

### 3.2 Weighted gene co-expression network analysis

To assess gene co-expression patterns further and identify modules associated with asthma, we performed WGCNA on the GSE137268 dataset. Hierarchical clustering of the samples and a trait heatmap confirmed the consistency of the sample grouping (Figure 2C). A soft-thresholding power of 12 was selected based on the scale-free topology criterion and mean connectivity (Figures 2D,E). Using the WGCNA package, a systematic clustering tree was constructed. In Figure 2F, each short vertical line represents a gene, and each color represents one module composed of genes with similar expression patterns. Three modules were identified (Figure 2G), among which the MEgrey module was most significantly associated with asthma (cor = 0.37, *p* < 0.001), as shown in the module-trait heatmap (Figure 2H). To refine potential diagnostic biomarkers, an integrative analysis was performed using the DEGs and the genes from the WGCNA grey modules. A total of 19 overlapping genes were identified and considered core asthma-related candidates (Figure 2I).

### 3.3 Identification of candidate genes via machine learning

**Figure 3:**
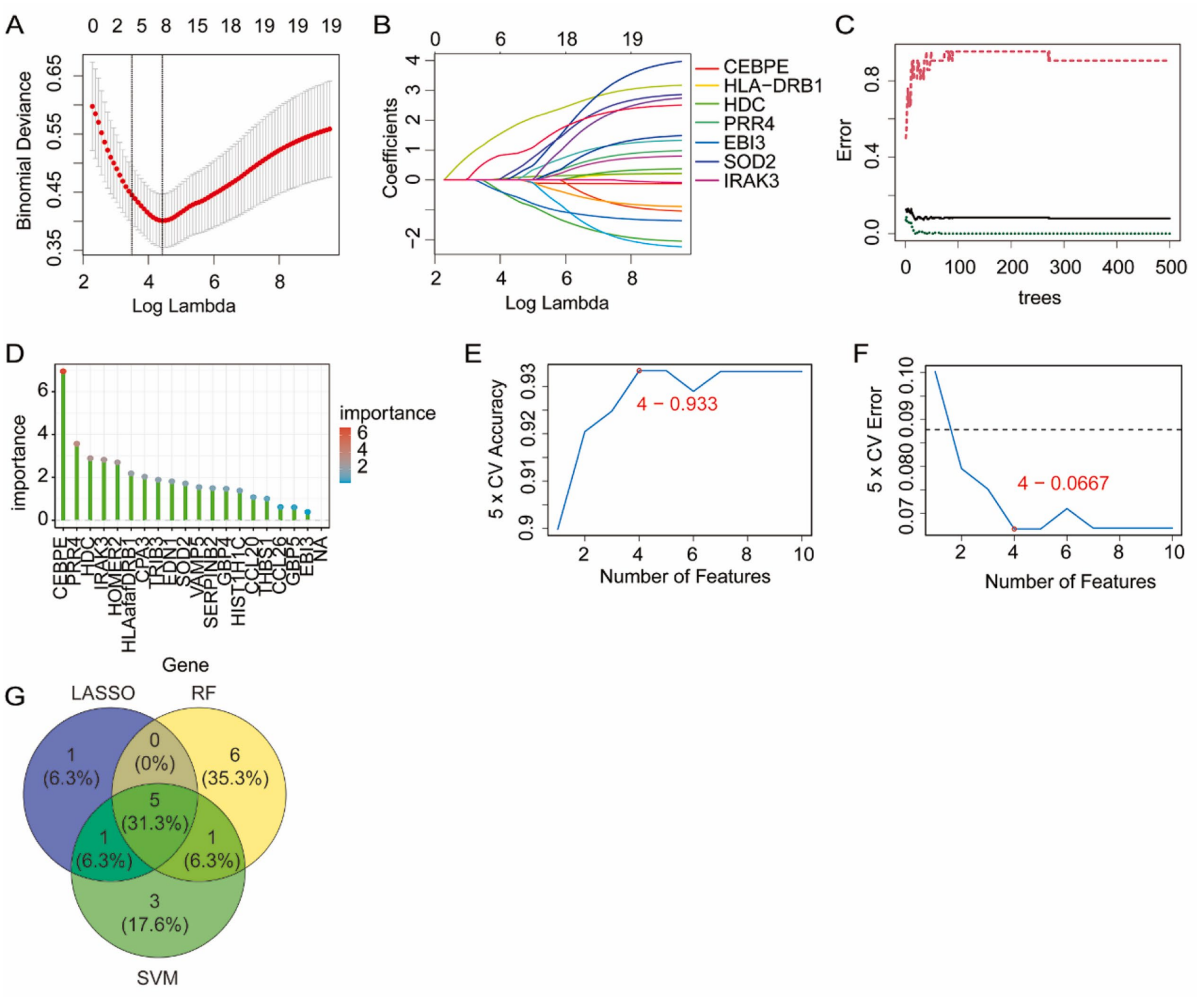
Candidate diagnostic biomarkers were identified via machine learning in the GSE134544 dataset. **(A)** Optimal lambda selection in LASSO regression. **(B)** LASSO coefficient profiles. **(C,D)** Random forest model: error rate plot and gene importance ranking. **(E,F)** SVM-RFE: cross-validation error and accuracy for different feature subsets. **(G)** The Venn diagram shows features selected by LASSO, RF, and SVM-RFE.

The 19 identified genes underwent machine learning-based feature selection using the GSE134544 dataset. To conduct LASSO regression analysis, the optimal lambda value was determined by 10-fold cross-validation (Figure 3A), resulting in the selection of 7 genes with non-zero coefficients (Figure 3B). These genes were considered to have the strongest penalized regression association with the status of asthma. In the RF model, variable importance was computed using the Gini index, and 12 genes with importance scores >1.5 were retained for further analysis (Figures 3C,D), representing the most informative predictors of the classification of asthma. For SVM-RFE, recursive feature elimination was performed via cross-validation with an accuracy-based ranking criterion, and the top 10 ranked genes with AvgRank<10 were selected as candidate features (Figures 3E,F). Furthermore, we performance ROC analysis for the machine learning models to demonstrate their predictive validity. The AUC of ROC analysis was 0.924 (0.869–0.978) for LASSO regression analysis, 1 (1−1) for RF model, and 0.912 (0.826–0.998) for SVM-RFE, which exceeded 0.5 (Supplementary Figure S1). Finally, five genes (CEBPE, HDC, IRAK3, PRR4, and SOD2) were identified as overlapping features across all three algorithms, as illustrated in the Venn diagram (Figure 3G), and were thus considered robust candidate biomarkers for asthma.

### 3.4 Gene set enrichment analysis of candidate biomarkers

**Figure 4:**
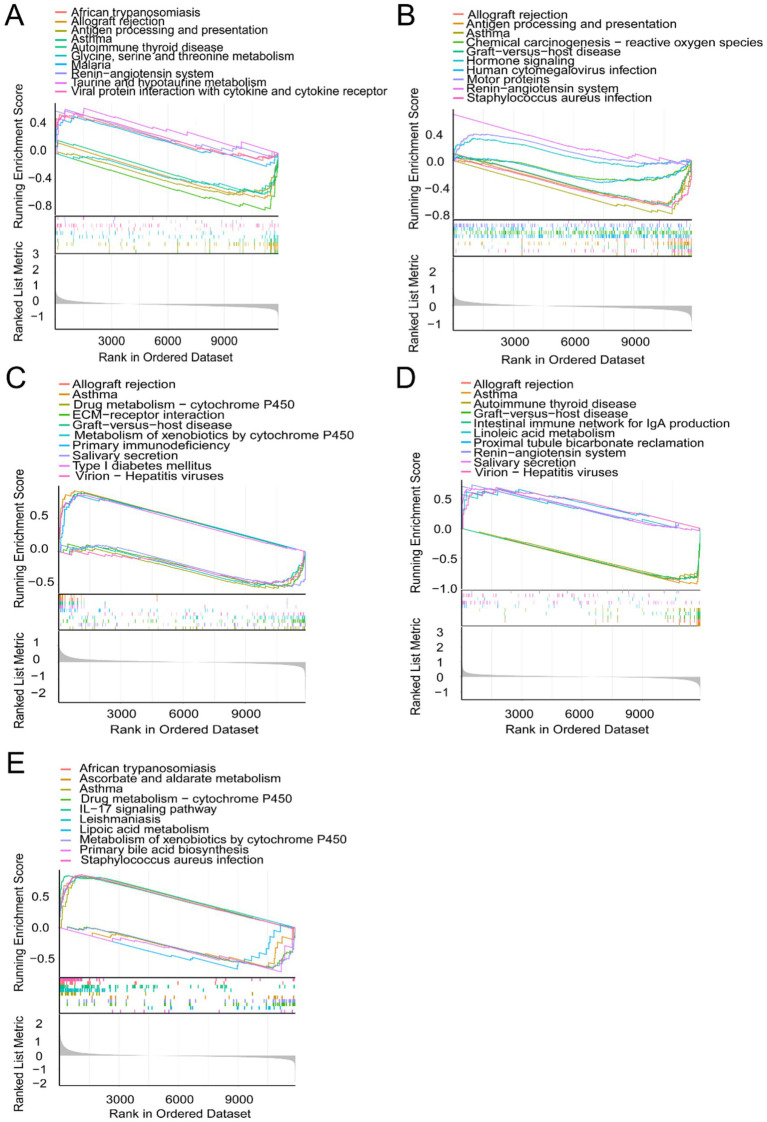
Gene set enrichment analysis (GSEA) of candidate diagnostic biomarkers in the GSE65204 dataset. **(A–E)** GSEA plots for CEBPE, HDC, IRAK3, PRR4, and SOD2, respectively. The samples were divided into high-expression and low-expression groups, and KEGG pathways were ranked based on enrichment scores. The top enriched pathways are indicated in each plot.

To elucidate the biological roles of the five candidate biomarkers (CEBPE, HDC, IRAK3, PRR4, and SOD2), GSEA was performed on the GSE65204 dataset. The analysis revealed that these genes were consistently enriched in several immune-related and metabolic pathways, highlighting their functional involvement in asthma pathogenesis. All genes were enriched in asthma, while most genes were involved in immune regulatory pathways and immune-related diseases, including allograft rejection, autoimmune thyroid disease, primary immunodeficiency, and graft-versus-host disease. We found that IRAK3 and SOD2 were enriched in the regulation of cytochrome P450. Most genes were linked primarily to metabolism, such as glycine, serine, and threonine metabolism; taurine and hypotaurine metabolism; and drug metabolism, such as cytochrome P450 (Figure 4). These findings collectively support the functional relevance of the five candidate genes and suggest that they may contribute to asthma.

### 3.5 Comparison of the baseline characteristics between the two groups

A total of 160 participants with asthma and 160 participants without asthma were screened and included. The two groups showed no significant differences in baseline characteristics, such as age structure, sex ratio, BMI, smoking history, education level, family history, or complications (all *p* > 0.05), indicating good comparability. The baseline characteristics of the two groups are presented in Table 2.

**Table 2:** Comparison of the general data between the two groups.

Factors	Indicator	Control group (*n* = 160)	Asthma group (*n* = 160)	χ^2^/*t*-value	*P*-value
Age (year)		55.11 ± 12.09	56.19 ± 11.71	0.812	0.418
Sex	Male	75 (46.875)	83 (51.875)	0.800	0.371
	Female	85 (53.125)	77 (48.125)		
BMI (kg/m^2^)		23.89 ± 2.62	23.33 ± 3.09	1.748	0.081
Moking history	Yes	50 (31.250)	55 (34.375)	0.354	0.552
	No	110 (68.750)	105 (65.625)		
Family history	Yes	33 (20.625)	21 (13.125)	3.208	0.073
	No	127 (79.375)	139 (86.875)		
Diabetes	Yes	79 (49.375)	92 (57.500)	2.123	0.145
	No	81 (50.625)	68 (42.500)		

### 3.6 Abundance of candidate genes in the blood

**Figure 5:**
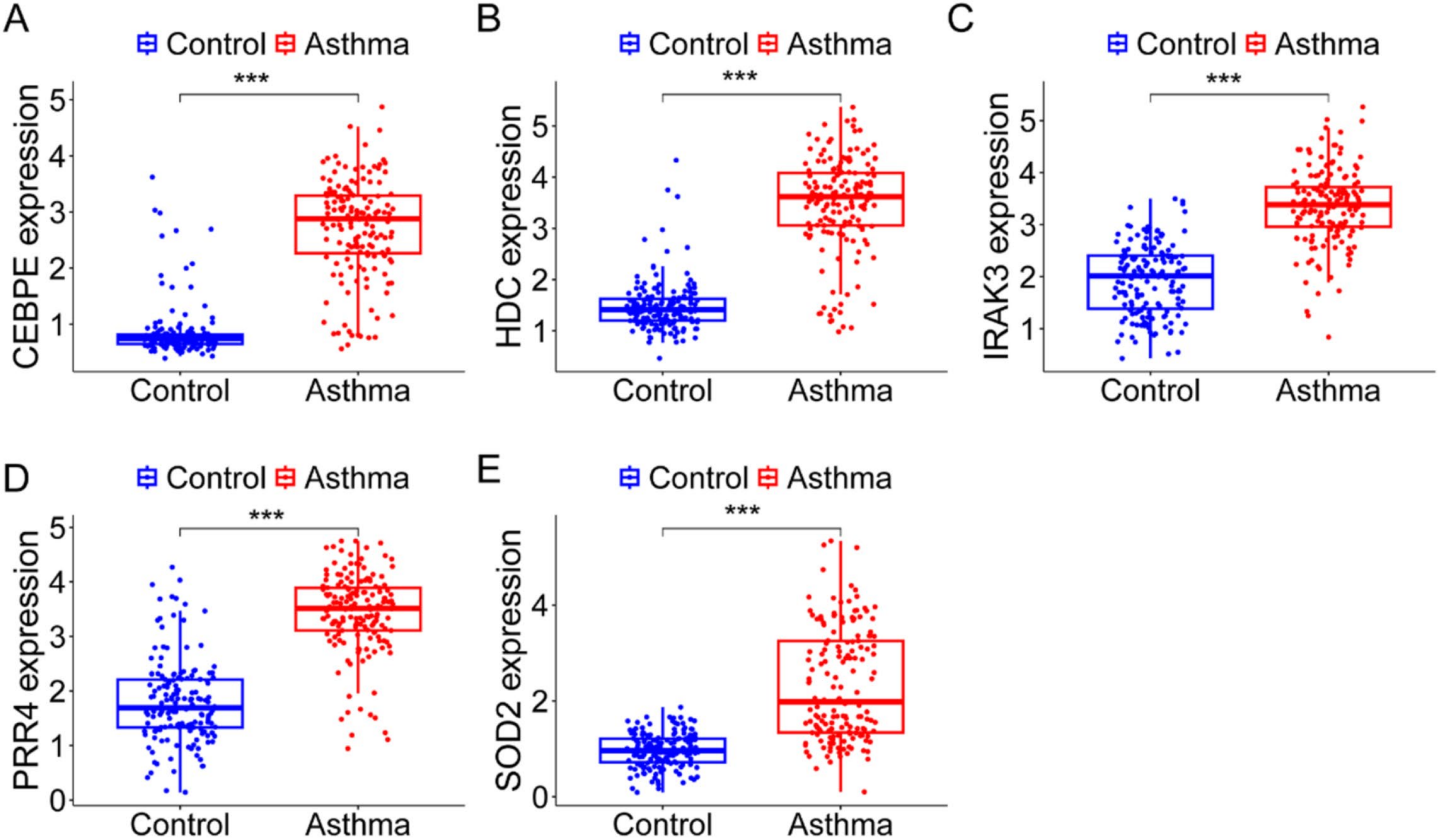
The abundance of genes in the serum of asthma patients and participants without asthma. The genes included CEBPE **(A)**, HDC **(B)**, PRR4 **(C)**, SOD2 **(D)**, and IRAK3 **(E)**. ***p < 0.001.

**Figure 6:**
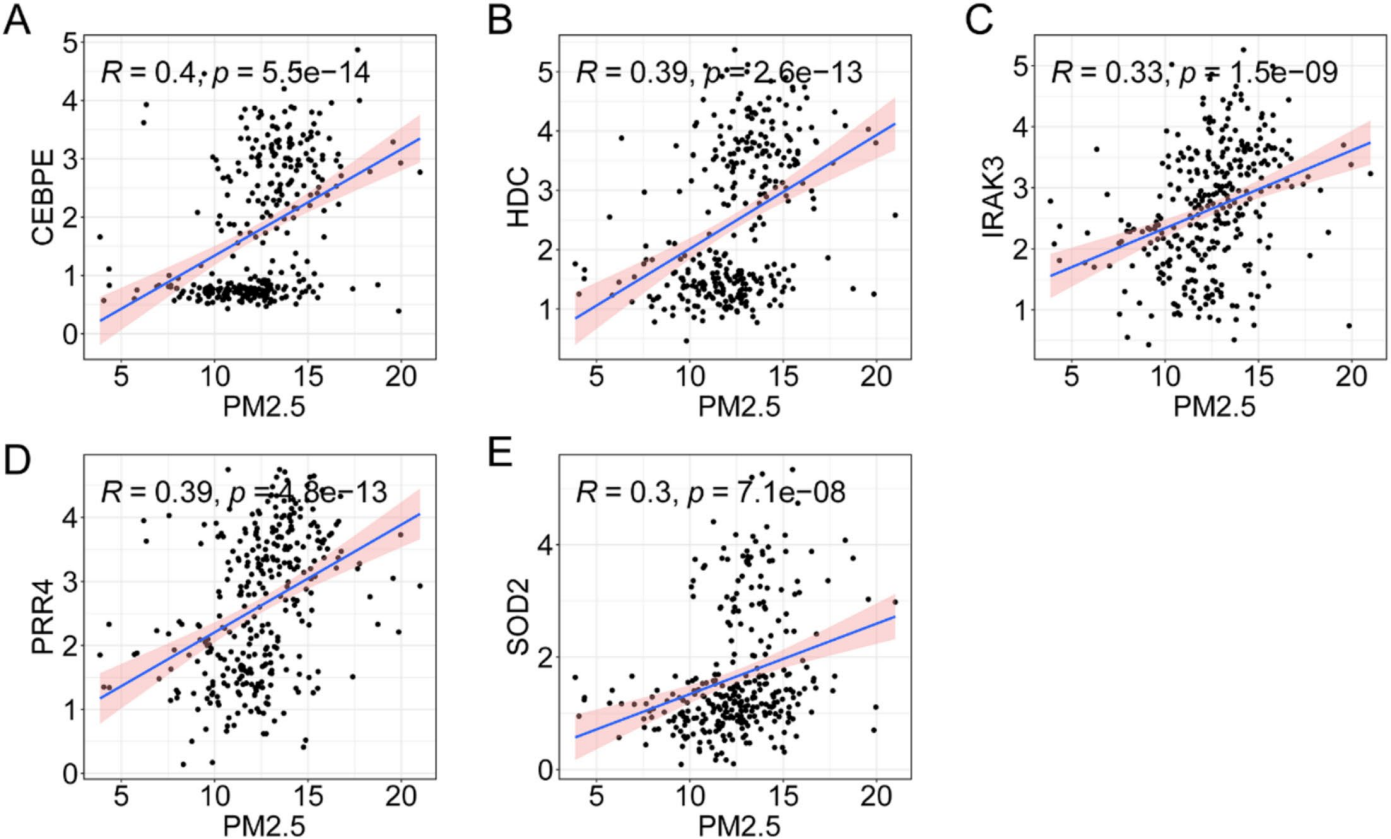
Relationships between genes and PM2.5 levels. The genes included CEBPE **(A)**, HDC **(B)**, PRR4 **(C)**, SOD2 **(D)**, and IRAK3 **(E)**.

To determine the clinical significance of the candidate genes, we collected blood from participants with/without asthma for validation via qRT-PCR. The results of qRT-PCR revealed that the expression of three genes of interest, including CEBP, was significantly greater in asthma patients than in participants without asthma (*p* < 0.001) (Figure 5A). The HDC levels in the serum of asthma patients were significantly greater than those in the serum of participants without asthma (*p* < 0.001, Figure 5B). Similar patterns were also observed for IRAK3, PRR4, and SOD2 (*p* < 0.001) (Figures 5C–E). The relationships between gene expression levels and PM2.5 levels were subsequently evaluated using the Pearson correlation coefficient. The results revealed a significant positive correlation between CEBPE and PM2.5 levels (Figure 6A). Similar patterns were found for HDC (Figure 6B), IRAK3 (Figure 6C), PRR4 (Figure 6D), and SOD2 (Figure 6E).

### 3.7 Univariate and multivariate logistic regression analyses

**Figure 7:**
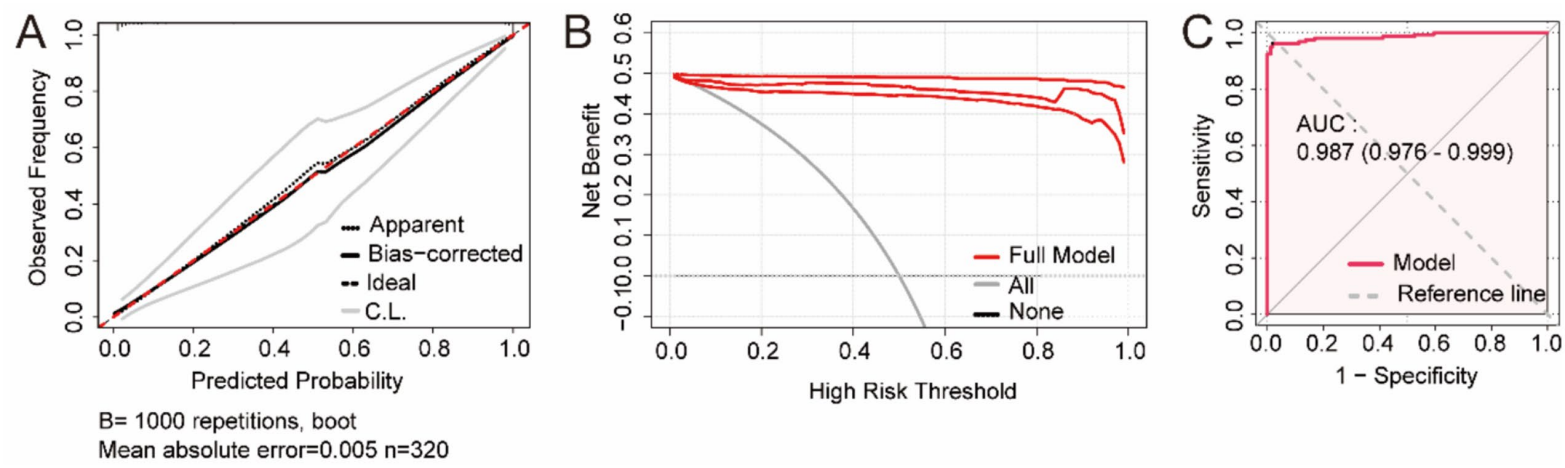
The performance indicators of the multivariate logistic regression model after adjusting for confounding factors, including **(A)** the calibration curves, **(B)** the DCA curves, and **(C)** the ROC curve.

Univariate and multivariate logistic regression analyses were conducted to assess the independent predictive role of these five genes for asthma. The presence or absence of asthma was considered to be the endpoint, and the selected geneswere included as independent variables in univariate logistic regression models. These five genes (CEBPE, HDC, IRAK3, PRR4, and SOD2) were significantly different (*p* < 0.05). The detailed results are presented in Table 3. To verify the relationship between these genes and asthma, multivariate logistic regression analysis was performed. The results confirmed that these five genes (CEBPE, HDC, IRAK3, PRR4, and SOD2) were independent risk factors for asthma, and the detailed results are presented in Table 4. After adjusting for confounding factors such as gender and age, these five genes (CEBPE, HDC, IRAK3, PRR4, and SOD2) were still independent risk factors for asthma (Table 5). Furthermore, the calibration curves of multivariate logistic regression model after adjusting for confounding factors in terms of the agreement between the observed outcomes and predicted probabilities (Figure 7A). While the DCA curve showed the large net benefits of the multivariate logistic regression model for predicting asthma (Figure 7B). The ROC curve indicated that the multivariate logistic regression model had a good distinguishing ability (Figure 7C). These findings suggest that the multivariate logistic regression model has a relatively high degree of accuracy.

**Table 3:** Results of univariate logistic regression analysis.

Factors	*β*	SE	Wald	*P*-value	Exp(*β*)	Exp(*β*) 95% CI	
						Down	Up
CEBPE	2.640	0.264	100.031	<0.001	14.018	8.355	23.517
HDC	2.470	0.249	98.412	<0.001	11.817	7.254	19.248
IRAK3	2.811	0.304	85.217	<0.001	16.620	9.151	30.185
PRR4	2.231	0.222	101.424	<0.001	9.311	6.031	14.374
SOD2	3.112	0.426	53.363	<0.001	22.470	9.749	51.790

**Table 4:** Results of multivariate logistic regression analysis.

Factors	*β*	SE	Wald	*P*-value	Exp(*β*)	Exp(*β*) 95% CI	
						Down	Up
CEBPE	0.940	0.417	5.086	0.024	2.560	1.131	5.793
HDC	0.860	0.435	3.915	0.048	2.364	1.008	5.544
IRAK3	0.918	0.417	4.855	0.028	2.504	1.107	5.665
PRR4	0.946	0.434	4.744	0.029	2.576	1.099	6.036
SOD2	3.204	0.928	11.922	0.001	24.629	3.996	151.797

**Table 5:** Results of multivariate logistic regression analysis after correcting for multiple confounding factors.

Factors	*β*	SE	Wald	*P*-value	Exp(*β*)	Exp(*β*) 95% CI	
						Down	Up
CEBPE	0.895	0.377	5.641	0.018	2.448	1.169	5.123
HDC	1.066	0.405	6.934	0.008	2.904	1.313	6.422
IRAK3	0.899	0.380	5.580	0.018	2.456	1.165	5.178
PRR4	1.000	0.436	5.271	0.022	2.719	1.158	6.387
SOD2	3.286	1.059	9.630	0.002	26.740	3.356	213.087

### 3.8 Significance test of the mediating effect

**Figure 8:**
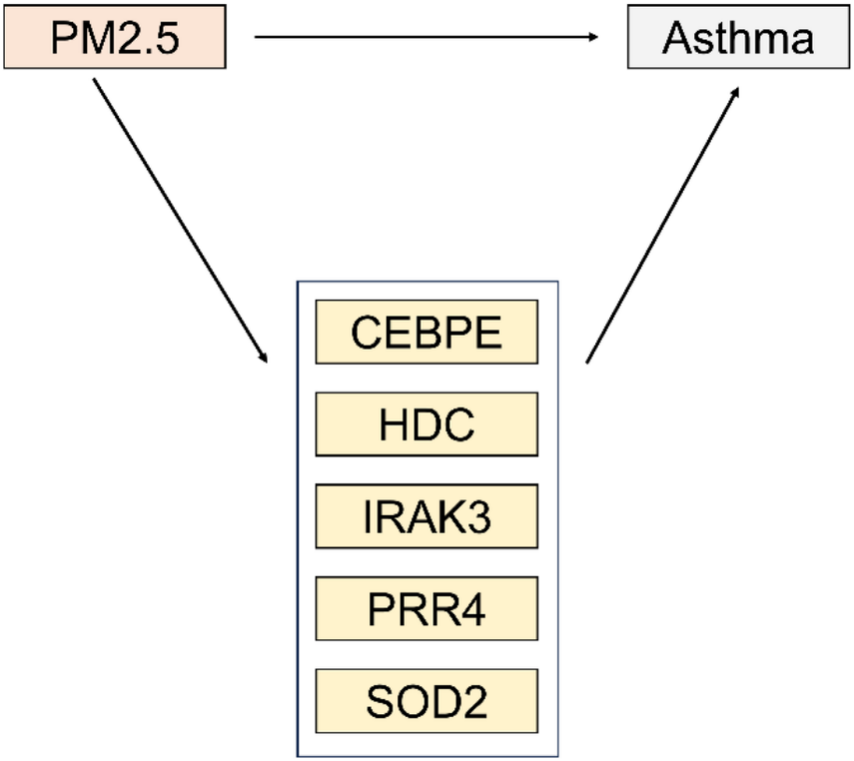
Relationships among PM2.5, latent variables, and asthma.

In this study, a mediation effect analysis was conducted to elucidate the potential mediating mechanisms between PM2.5 and the onset of asthma, involving several potential mediators, including CEBPE, HDC, IRAK3, PRR4, and SOD2. The results of the analysis indicated that partial mediation effects were observed in all mediation paths. For the path through which PM2.5 affects asthma through CEBPE, the indirect effect was 0.022, with a 95% confidence interval (CI) of (0.068, 0.161), a standard error (SE) of 0.024, a z/t value of 0.909, and a *p*-value of 0.363. Similarly, for the path where PM2.5 affects asthma through HDC, the indirect effect was 0.02 [95% CI (0.063, 0.148), *p* = 0.374], with an SE of 0.022 and a z/t value of 0.89. For the path where PM2.5 affects asthma through IRAK3, the indirect effect was 0.011 [95% CI (0.028, 0.085), *p* = 0.46], with an SE of 0.014 and a z/t value of 0.739. For the path where PM2.5 affects asthma through PRR4, the indirect effect was 0.012 [95% CI (0.033, 0.1), *p* = 0.475], with an SE of 0.017 and a z/t value of 0.714. Finally, for the path where PM2.5 affects asthma through SOD2, the indirect effect was 0.01 [95% CI (0.028, 0.074), *p* = 0.419], with an SE of 0.012 and a z/t value of 0.808 (Table 6 and Figure 8). These findings suggest that these variables may play a partial mediating role in the process by which PM2.5 affects the onset of asthma, providing important clues and serving as a reference for further in-depth research on the internal mechanisms by which PM2.5 induces asthma.

**Table 6:** Bootstrap test of the mediating effect.

Item	ACME_estimate	ACME_*P-value*	ACME_CI	ADE_estimate	ADE_*P-value*	Total_effect	Prop_mediated	Mediation_type
PM2.5= > CEBPE= > Asthma	0.288	<0.001	[0.214, 0.376]	0.209	<0.001	0.497	0.58	Partial mediation
PM2.5= > HDC= > Asthma	0.283	<0.001	[0.215, 0.360]	0.214	<0.001	0.497	0.569	Partial mediation
PM2.5= > PRR4= > Asthma	0.248	<0.001	[0.186, 0.321]	0.249	<0.001	0.497	0.5	Partial mediation
PM2.5= > IRAK3= > Asthma	0.202	<0.001	[0.144, 0.266]	0.295	<0.001	0.497	0.406	Partial mediation
PM2.5= > SOD2= > Asthma	0.153	<0.001	[0.108, 0.197]	0.344	<0.001	0.497	0.307	Partial mediation

## 4 Discussion

With rapid industrialization and urbanization, the deterioration of air quality not only poses a threat to the health of the general population but also exacerbates the challenges faced by asthma patients, making their situation even more severe (18, 19). Studies have reported that prolonged exposure to polluted environments not only increases the incidence rate of asthma but also intensifies the symptoms of patients and increases the frequency of attacks (20). For every 10 μg increase in the PM2.5 concentration/m3 of air, there is a 3.5% increase in hospitalization rates among asthma patients (21, 22). Therefore, we aimed to identify the mediating molecules associated with air pollution and the incidence of asthma. Using GEO datasets, 715 DEGs were identified, including 363 upregulated and 352 downregulated genes. WGCNA revealed that the MEgrey module was most significantly related to asthma. A total of 19 overlapping genes were identified and subjected to machine learning-based feature selection. Finally, five hub genes (CEBPE, HDC, IRAK3, PRR4, and SOD2) were recognized as overlapping features across all three algorithms, including LASSO regression, RF, and SVM-RFE. The results of the univariate and multivariate logistic regression analyses revealed that CEBPE, HDC, IRAK3, PRR4, and SOD2 were independent risk factors. A mediation effect analysis was conducted, and the results revealed that these five genes played a mediating role in the PM2.5 and onset of asthma.

Some studies have indicated that, besides external environmental factors, genetics (especially key genes) may play an important role in the pathogenesis of asthma (23, 24). With the advancement of gene chip technology in recent years, a large amount of sequencing data on disease onset and progression has been uploaded to gene expression databases (25). Investigating key genes to elucidate the molecular mechanisms underlying the development and progression of asthma has become a research hotspot in recent years (26). Some studies have used bioinformatics analysis methods to examine gene chips from bronchial epithelial samples of severe asthma patients and healthy controls, identifying key DEGs and elucidating their roles in major signaling pathways during severe asthma onset (27, 28). However, these studies did not address whether the DEGs are associated with the relationship between the pathogenesis of asthma and air pollution. To bridge this gap, we analyzed the mediating effects of these DEGs between the onset of asthma and air pollution. By conducting differential gene expression analysis and WGCNA, we obtained 18 asthma-related hub genes from two GEO datasets. We subsequently conducted machine learning analysis on these 19 genes using three methods to further narrow the scope of asthma-related hub genes. Five genes (CEBPE, HDC, IRAK3, PRR4, and SOD2) were identified as overlapping features through LASSO regression, RF, and SVM-RFE. CEBP belongs to the C/EBP family and participates in the development of inflammatory diseases. Yamini V Virkud found a correlation between CEBP and immunoglobulin E levels in the “Genetics of Asthma in Costa Rica Study” and “Childhood Asthma Management Program” cohorts. Their findings indicated that CEBP may be involved in the molecular mechanism of asthma development (29). Some studies have found that HDC is involved in the regulation of goblet cell hyperplasia in allergic airway inflammation (30, 31). Through microarray screening and subsequent validation, Winter et al. reported that HDC levels were higher in eosinophilic asthma patients than in non-eosinophilic asthma patients (32), which agrees with our findings. Zhong revealed that PRR4 is a specific asthma gene through bioinformatics approaches (33). This observation is consistent with our data. Through Mendelian randomization analysis, previous studies have shown that SOD is involved in nasal mucosal biology mediated by air pollutants (34). SOD2 is correlated with a lower risk of childhood bronchial asthma (35). Consistent with our results, through machine learning, Zhang discovered that SOD is a mitochondria-related biomarker with diagnostic value for neutropenic asthma (36). Genetic variants of SOD2, which can reflect oxidative stress metabolism, might modify the association of phthalate exposure with asthma (37). IRAK3 encodes a protein that negatively regulates Toll-like receptor signaling pathways involved in innate host defense and in the control of adaptive immune responses (38). Significantly greater expression of IRAK3 was found in healthy individuals, patients with chronic persistent asthma, and patients with acute exacerbation of asthma (39). Therefore, the identification of these genes provides new insights into the molecular mechanisms underlying air pollution-induced asthma and may serve as biomarkers for the prevention and treatment of asthma. GSEA revealed that these genes are involved in the development of asthma, antigen processing and presentation, and IgA production. These findings further suggest that these genes may be involved in the occurrence of asthma caused by environmental pollution.

To determine the role of these key genes in the occurrence of asthma caused by environmental pollution, we provided strong evidence that the expression of genes of interest (CEBPE, HDC, IRAK3, PRR4, and SOD2) increased significantly in 160 participants with asthma. This finding was also similar to the results of multiple regression analysis, in which five genes (CEBPE, HDC, IRAK3, PRR4, and SOD2) were found to be independent risk factors for asthma. After adjusting for gender and age, these five genes were still independent risk factors for asthma. Importantly, the results of DCA curve, ROC curve, and calibration curves indicate that the multivariate logistic regression model is reliable and trustworthy. Considering the significant linear correlation between the levels of these genes and PM2.5 levels, we speculate that these genes mediate the onset of asthma induced by environmental pollution. We conducted a mediation effect analysis to determine whether these genes are mediators of between PM2.5 and the onset of asthma. The results revealed that five genes of interest (CEBPE, HDC, IRAK3, PRR4, and SOD2) showed a strong mediating effect between PM2.5 and the onset of asthma, suggesting that PM2.5 may alter the expression of these genes, thereby causing the onset of asthma. We speculate that these changes may be attributed to the regulatory effects of these genes on immune signaling pathways, inflammation, etc.; GSEA revealed these regulatory effects. Moreover, CEBPE is involved in myeloid cell development and the induction of several inflammatory mediators. Knocking down CEBPE leads to the failure of functional neutrophil and eosinophil production, impairing chemotaxis and bactericidal activity (40). HDC, a histamine-producing enzyme, is involved in the late and chronic phases of allergic and nonallergic inflammation (41). An increase in HDC levels promotes the occurrence of asthma. IRAK3 plays a key role in maintaining chronic Th2 airway inflammation via the inhibition of lung dendritic cell-mediated activation of Th1 (42). PRR4 is the most important gene in regulating the Th2 endotype and exhibits the strongest correlation with resting mast cells (43). Many studies have confirmed that SOD2 regulates allergic lung inflammation by regulating the production of mitochondrial ROS (44, 45). These studies suggest that environmental air pollution may influence the risk of asthma by modulating the expression of these genes. This discovery provides new clues to better understand how air pollution affects respiratory health and informs strategies for the prevention and treatment of asthma.

This study had some limitations. First, although this study included 320 participants, the sample size may be relatively small for a specific region or the entire asthma-afflicted population, thereby limiting the generalizability and representativeness of the results. Second, although the clinical validation cohort was large enough, the participants were recruited from a single city within the population of a specific region. The differences in air pollution components and levels among different regions may limit the extrapolation of these findings to other areas. Therefore, conducting multi-center research in the future is necessary. The differences in exposure caused by factors such as occupation, commuting methods, and the use of indoor air purification equipment among individuals can lead to non-differential misclassification bias. Further in-depth analysis will still be necessary to determine the environmental pollution experienced by individuals during the exposure period, and thereby identify the mediating factors. Finally, in biological processes, complex interactions might exist between genes, and these factors were not fully considered in this study, which might have introduced biases into the results. Therefore, in-depth molecular mechanism research in the future will help explain the role of these factors between onset of asthma and PM2.5.

## 5 Conclusion

By conducting bioinformatics analysis, we identified five genes (CEBPE, HDC, IRAK3, PRR4, and SOD2) associated with asthma. This study provides strong evidence for the mediating effect of these genes on the association between PM2.5 and the onset of asthma. These findings not only enrich our understanding of the etiology of air pollution and the onset of asthma but also provide new information for the prevention and treatment of asthma in the future.

## Data Availability

The original contributions presented in the study are included in the article/Supplementary material, further inquiries can be directed to the corresponding author.

## Footnotes

1
https://www.ncbi.nlm.nih.gov/geo/

## References

[1] Lommatzsch M Brusselle GG Levy ML Canonica GW Pavord ID Schatz M . A(2)BCD: a concise guide for asthma management. Lancet Respir Med. (2023) 11:573–6. doi: 10.1016/s2213-2600(22)00490-8, 36716752

[2] Wan R Srikaram P Guntupalli V Hu C Chen Q Gao P. Cellular senescence in asthma: from pathogenesis to therapeutic challenges. EBioMedicine. (2023) 94:104717. doi: 10.1016/j.ebiom.2023.104717, 37442061 PMC10362295

[3] Xing Y Leung AS Wong GW. From preschool wheezing to asthma: environmental determinants. Pediatr Allergy Immunol. (2023) 34:e14049. doi: 10.1111/pai.14049, 38010001

[4] Melén E Zar HJ Siroux V Shaw D Saglani S Koppelman GH . Asthma inception: epidemiologic risk factors and natural history across the life course. Am J Respir Crit Care Med. (2024) 210:737–54. doi: 10.1164/rccm.202312-2249SO, 38981012 PMC11418887

[5] Liu K Hua S Song L. PM2.5 exposure and asthma development: the key role of oxidative stress. Oxidative Med Cell Longev. (2022) 2022:3618806. doi: 10.1155/2022/3618806, 35419163 PMC9001082

[6] Agache I Canelo-Aybar C Annesi-Maesano I Cecchi L Rigau D Rodríguez-Tanta LY . The impact of outdoor pollution and extreme temperatures on asthma-related outcomes: a systematic review for the EAACI guidelines on environmental science for allergic diseases and asthma. Allergy. (2024) 79:1725–60. doi: 10.1111/all.16041, 38311978

[7] Cheng PP Yu F Chen SJ Feng X Jia ZH Hu SH . PM2.5 exposure-induced senescence-associated secretory phenotype in airway smooth muscle cells contributes to airway remodeling. Environ Pollut. (2024) 347:123674. doi: 10.1016/j.envpol.2024.123674, 38458517

[8] Piao CH Fan Y Nguyen TV Song CH Kim HT Chai OH. PM2.5 exposure regulates Th1/Th2/Th17 cytokine production through NF-κB signaling in combined allergic rhinitis and asthma syndrome. Int Immunopharmacol. (2023) 119:110254. doi: 10.1016/j.intimp.2023.110254, 37163921

[9] Bryan L Landrigan P. PM (2.5) pollution in Texas: a geospatial analysis of health impact functions. Front Public Health. (2023) 11:1286755. doi: 10.3389/fpubh.2023.1286755, 38106908 PMC10722416

[10] Zhang Y Yin X Zheng X. The relationship between PM2.5 and the onset and exacerbation of childhood asthma: a short communication. Front Pediatr. (2023) 11:1191852. doi: 10.3389/fped.2023.1191852, 37593445 PMC10429171

[11] Mostafavi N Jeong A Vlaanderen J Imboden M Vineis P Jarvis D . The mediating effect of immune markers on the association between ambient air pollution and adult-onset asthma. Sci Rep. (2019) 9:8818. doi: 10.1038/s41598-019-45327-4, 31217483 PMC6584571

[12] Andreasson LM Dyhre-Petersen N Hvidtfeldt M Jørgensen G Von Bülow A Klein DK . Airway hyperresponsiveness correlates with airway TSLP in asthma independent of eosinophilic inflammation. J Allergy Clin Immunol. (2024) 153:988–997.e11. doi: 10.1016/j.jaci.2023.11.915, 38081546

[13] Szefler S Corren J Silverberg JI Okragly A Sun Z Natalie CR . Lebrikizumab decreases type 2 inflammatory biomarker levels in patients with asthma: data from randomized phase 3 trials (LAVOLTA I and II). Immunotherapy. (2024) 16:1211–6. doi: 10.1080/1750743x.2024.2439777, 39781908 PMC11759530

[14] Altman MC Kattan M O'Connor GT Murphy RC Whalen E LeBeau P . Associations between outdoor air pollutants and non-viral asthma exacerbations and airway inflammatory responses in children and adolescents living in urban areas in the USA: a retrospective secondary analysis. Lancet Planet Health. (2023) 7:e33–44. doi: 10.1016/s2542-5196(22)00302-3, 36608946 PMC9984226

[15] Society AgoCT. Guidelines for bronchial asthma prevent and management (2020 edition) asthma group of Chinese Throacic society. Zhonghua Jie He He Hu Xi Za Zhi. (2020) 43:1023–48. doi: 10.3760/cma.j.cn112147-20200618-00721, 33333637

[16] Zhao X Shen Z Han F Bharti B Feng S Du J . Pollution characteristics and health risk assessment of heavy metals in PM2.5 in Fuxin, China. Environ Geochem Health. (2024) 46:511. doi: 10.1007/s10653-024-02275-x, 39527341 PMC11554730

[17] Livak KJ Schmittgen TD. Analysis of relative gene expression data using real-time quantitative PCR and the 2− ΔΔCT method. Methods. (2001) 25:402–8. doi: 10.1006/meth.2001.1262 11846609

[18] Maung TZ Bishop JE Holt E Turner AM Pfrang C. Indoor air pollution and the health of vulnerable groups: a systematic review focused on particulate matter (PM), volatile organic compounds (VOCs) and their effects on children and people with pre-existing lung disease. Int J Environ Res Public Health. (2022) 19:8752. doi: 10.3390/ijerph19148752, 35886604 PMC9316830

[19] Marcot C Migueres N Ott M Khayath N De Blay F. Allergenic and chemical pollutants of indoor environments and asthma: characterization, assessment and eviction. Rev Mal Respir. (2023) 40:630–45. doi: 10.1016/j.rmr.2023.06.001, 37391338

[20] Pedersen M Liu S Zhang J Jovanovic Andersen Z Brandt J Budtz-Jørgensen E . Early-life exposure to ambient air pollution from multiple sources and asthma incidence in children: a Nationwide birth cohort study from Denmark. Environ Health Perspect. (2023) 131:57003. doi: 10.1289/ehp11539, 37162236 PMC10171081

[21] Varghese D Ferris K Lee B Grigg J Pinnock H Cunningham S. Outdoor air pollution and near-fatal/fatal asthma attacks in children: a systematic review. Pediatr Pulmonol. (2024) 59:1196–206. doi: 10.1002/ppul.26932, 38477643

[22] Huang J Yang X Fan F Hu Y Wang X Zhu S . Outdoor air pollution and the risk of asthma exacerbations in single lag0 and lag1 exposure patterns: a systematic review and meta-analysis. J Asthma. (2022) 59:2322–39. doi: 10.1080/02770903.2021.2008429, 34809505

[23] Haider S Simpson A Custovic A. Genetics of asthma and allergic diseases. Handb Exp Pharmacol. (2022) 268:313–29. doi: 10.1007/164_2021_484, 34085121

[24] Wolters AAB Kersten ETG Koppelman GH. Genetics of preschool wheeze and its progression to childhood asthma. Pediatr Allergy Immunol. (2024) 35:e14067. doi: 10.1111/pai.14067, 38284918

[25] Liu T Woodruff PG Zhou X. Advances in non-type 2 severe asthma: from molecular insights to novel treatment strategies. Eur Respir J. (2024) 64:2300826. doi: 10.1183/13993003.00826-2023, 38697650 PMC11325267

[26] Jia Y Wang H Ma B Zhang Z Wang J Wang J . Lipid metabolism-related genes are involved in the occurrence of asthma and regulate the immune microenvironment. BMC Genomics. (2024) 25:129. doi: 10.1186/s12864-023-09795-3, 38297226 PMC10832186

[27] Ooka T Zhu Z Liang L Celedon JC Harmon B Hahn A . Integrative genetics-metabolomics analysis of infant bronchiolitis-childhood asthma link: a multicenter prospective study. Front Immunol. (2022) 13:1111723. doi: 10.3389/fimmu.2022.1111723 36818476 PMC9936313

[28] Makrinioti H Morita H Nastouli E Jartti T. Editorial: bridging the gap between immunology, virology, genetics, and epigenetics in bronchiolitis: the multiomics pathway to asthma development. Front Immunol. (2023) 14:1154121. doi: 10.3389/fimmu.2023.1154121, 36895569 PMC9989251

[29] Virkud YV Kelly RS Croteau-Chonka DC Celedón JC Dahlin A Avila L . Novel eosinophilic gene expression networks associated with IgE in two distinct asthma populations. Clin Exp Allergy. (2018) 48:1654–64. doi: 10.1111/cea.13249, 30107053 PMC6659730

[30] Yamauchi K Piao HM Nakadate T Shikanai T Nakamura Y Ito H . Enhanced goblet cell hyperplasia in HDC knockout mice with allergic airway inflammation. Allergol Int. (2009) 58:125–34. doi: 10.2332/allergolint.O-08-547, 19153539

[31] Sziksz E Tibor Kozma G Komlósi ZI Pállinger E Kardos M Szebeni B . Increased synthesis of vascular endothelial growth factor in allergic airway inflammation in histidine decarboxylase knockout (HDC(−/−)) mice. Exp Lung Res. (2010) 36:420–30. doi: 10.3109/01902141003767955, 20715981

[32] Winter NA Qin L Gibson PG McDonald VM Baines KJ Faulkner J . Sputum mast cell/basophil gene expression relates to inflammatory and clinical features of severe asthma. J Allergy Clin Immunol. (2021) 148:428–38. doi: 10.1016/j.jaci.2021.01.033, 33609626

[33] Zhong X Song J Lei C Wang X Wang Y Yu J . Machine learning-based screening of asthma biomarkers and related immune infiltration. Front Allergy. (2025) 6:1506608. doi: 10.3389/falgy.2025.1506608, 39963184 PMC11831286

[34] Irizar H Chun Y Hsu HL Li YC Zhang L Arditi Z . Multi-omic integration reveals alterations in nasal mucosal biology that mediate air pollutant effects on allergic rhinitis. Allergy. (2024) 79:3047–61. doi: 10.1111/all.16174, 38796780 PMC11560721

[35] Anber NH Ahmed Shahin HE Badawy HK Oraby EA Mohammed SA Shaaban EIA . Potential impact of SOD2 (rs4880; p.Val16Ala) variant with the susceptibility for childhood bronchial asthma. Biochem Genet. (2025) 63:789–816. doi: 10.1007/s10528-024-10742-4, 38522064

[36] Lin L Liao ZH Li CQ. Insight into the role of mitochondrion-related gene anchor signature in mitochondrial dysfunction of neutrophilic asthma. J Asthma. (2024) 61:912–29. doi: 10.1080/02770903.2024.2311241, 38294718

[37] Wang IJ Karmaus WJ. Oxidative stress-related genetic variants may modify associations of phthalate exposures with asthma. Int J Environ Res Public Health. (2017) 14:14. doi: 10.3390/ijerph14020162, 28208751 PMC5334716

[38] Pino-Yanes M Sánchez-Machín I Cumplido J Figueroa J Torres-Galván MJ González R . IL-1 receptor-associated kinase 3 gene (IRAK3) variants associate with asthma in a replication study in the Spanish population. J Allergy Clin Immunol. (2012) 129:573–5. doi: 10.1016/j.jaci.2011.10.001, 22070913

[39] Wei W Huang J Ma Y Ma X Fang L Fang W . IL-1 signaling pathway molecules as key markers in childhood asthma. Pediatr Allergy Immunol. (2021) 32:305–13. doi: 10.1111/pai.13388, 33025692

[40] Shyamsunder P Shanmugasundaram M Mayakonda A Dakle P Teoh WW Han L . Identification of a novel enhancer of CEBPE essential for granulocytic differentiation. Blood. (2019) 133:2507–17. doi: 10.1182/blood.2018886077, 30952671

[41] Hirasawa N. Expression of histidine decarboxylase and its roles in inflammation. Int J Mol Sci. (2019) 20:20. doi: 10.3390/ijms20020376, 30654600 PMC6359378

[42] Liu Y Zhang M Lou L Li L Zhang Y Chen W . IRAK-M associates with susceptibility to adult-onset asthma and promotes chronic airway inflammation. J Immunol. (2019) 202:899–911. doi: 10.4049/jimmunol.1800712, 30617222 PMC6344301

[43] Gong R Wang Z Tan G Huang Y. Bioinformatics analysis revealed underlying molecular mechanisms associated with asthma severity and identified GABAergic related pathway as a potential therapy for Th2-high endotype asthma. Heliyon. (2024) 10:e28401. doi: 10.1016/j.heliyon.2024.e28401, 38586354 PMC10998110

[44] Seo YS Kim HS Lee AY Chun JM Kim SB Moon BC . Codonopsis lanceolata attenuates allergic lung inflammation by inhibiting Th2 cell activation and augmenting mitochondrial ROS dismutase (SOD2) expression. Sci Rep. (2019) 9:2312. doi: 10.1038/s41598-019-38782-6, 30783201 PMC6381190

[45] Chen ML Zhu XH Ran L Lang HD Yi L Mi MT. Trimethylamine-N-oxide induces vascular inflammation by activating the NLRP3 Inflammasome through the SIRT3-SOD2-mtROS signaling pathway. J Am Heart Assoc. (2017) 6:6. doi: 10.1161/jaha.117.006347, 28871042 PMC5634285

