# Supplementary figures and images for: Screening and identification of key serum biomarkers between PM2.5 and-induced asthma onset
Source: Front Public Health. 2026 Jan 12;13:1693575. doi: 10.3389/fpubh.2025.1693575 (PMC12833453; doi:10.3389/fpubh.2025.1693575)

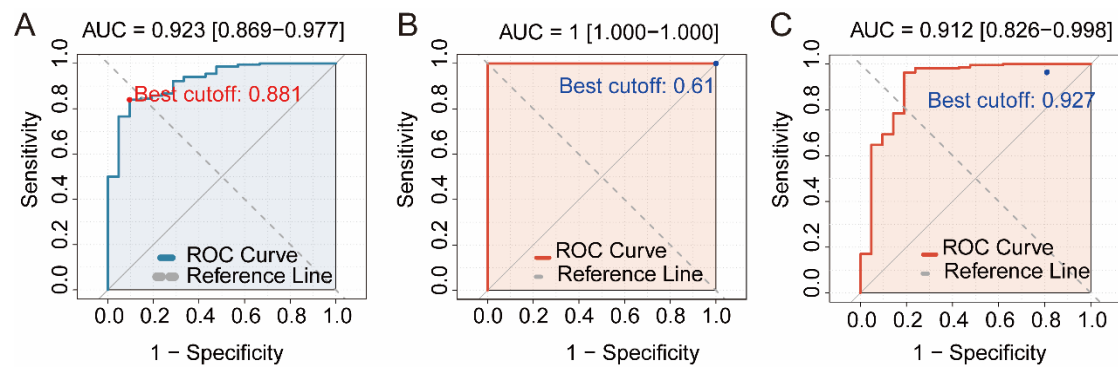

**Figures S1.** The AUC of ROC analysis for LASSO (A), RF (B), and SVM-RFE (C).

Supplement: Supplementary file 1 [file Image_1.pdf]
